# The Effects of Dietary Advanced Glycation End-Products on Neurocognitive and Mental Disorders

**DOI:** 10.3390/nu14122421

**Published:** 2022-06-10

**Authors:** Nathan M. D’Cunha, Domenico Sergi, Melissa M. Lane, Nenad Naumovski, Elizabeth Gamage, Anushri Rajendran, Matina Kouvari, Sarah Gauci, Thusharika Dissanayka, Wolfgang Marx, Nikolaj Travica

**Affiliations:** 1 Discipline of Nutrition and Dietetics, Faculty of Health, University of Canberra, Canberra, ACT 2601, Australianenad.naumovski@canberra.edu.au (N.N.); matina.kouvari@canberra.edu.au (M.K.); 2 Functional Foods and Nutrition Research (FFNR) Laboratory, University of Canberra, Bruce, ACT 2617, Australia; 3 Department of Translational Medicine, University of Ferrara, 44121 Ferrara, Italy; domenico.sergi@unife.it; 4 Food and Mood Centre, IMPACT—The Institute for Mental and Physical Health and Clinical Translation, School of Medicine, Barwon Health, Deakin University, Geelong, VIC 3220, Australia; melissalane0080@gmail.com (M.M.L.); egamage@deakin.edu.au (E.G.); a.rajendran@deakin.edu.au (A.R.); t.dissanayaka@deakin.edu.au (T.D.); wolf.marx@deakin.edu.au (W.M.); 5 Department of Nutrition-Dietetics, Harokopio University, 17671 Athens, Greece; 6 Institute for Intelligent Systems Research and Innovation (IISRI), Deakin University, Waurn Ponds, VIC 3216, Australia; 7 Centre for Human Psychopharmacology, Swinburne University, Melbourne, VIC 3122, Australia; sarah.gauci@deakin.edu.au; 8 Heart and Mind Research, IMPACT, Institute for Innovation in Physical and Mental Health and Clinical Translation, School of Medicine, Deakin University, Geelong, VIC 3220, Australia

**Keywords:** AGE, neurodegenerative diseases, cognitive function, dementia, depression, mental health, diet, nutritional psychiatry

## Abstract

Advanced glycation end products (AGEs) are glycated proteins or lipids formed endogenously in the human body or consumed through diet. Ultra-processed foods and some culinary techniques, such as dry cooking methods, represent the main sources and drivers of dietary AGEs. Tissue accumulation of AGEs has been associated with cellular aging and implicated in various age-related diseases, including type-2 diabetes and cardiovascular disease. The current review summarizes the literature examining the associations between AGEs and neurocognitive and mental health disorders. Studies indicate that elevated circulating AGEs are cross-sectionally associated with poorer cognitive function and longitudinally increase the risk of developing dementia. Additionally, preliminary studies show that higher skin AGE accumulation may be associated with mental disorders, particularly depression and schizophrenia. Potential mechanisms underpinning the effects of AGEs include elevated oxidative stress and neuroinflammation, which are both key pathogenetic mechanisms underlying neurodegeneration and mental disorders. Decreasing dietary intake of AGEs may improve neurological and mental disorder outcomes. However, more sophisticated prospective studies and analytical approaches are required to verify directionality and the extent to which AGEs represent a mediator linking unhealthy dietary patterns with cognitive and mental disorders.

## 1. Introduction

With global rates of cognitive impairment and mental disorders on the rise, the development of effective preventative and treatment measures is of paramount importance [1,2]. Cognitive impairment results from a complex interplay of many factors, including age, socioeconomic status, and environmental factors [3]. Diet is emerging as a key bidirectional factor in shaping cognitive and mental health trajectories [3,4,5]. In particular, the Western-style dietary pattern, which is typically high in consumption of ultra-processed foods that contain excess sugar, salt, saturated and trans fats, refined grains and energy, has been associated with cognitive impairment and depression [6,7]. Dietary advanced glycation end products (AGEs) have been touted as potential mediators on the causal pathway from the Western diet to brain health. Dietary AGEs are abundant in highly processed foods, synthesized via non-enzymatic reactions triggered by dry heat cooking methods [8], and are formed endogenously in response to hyperglycaemia and consumption of fructose [9]. 

Over decades, AGEs accumulate at the cellular level in body tissue, where they have the potential to irreversibly increase the rate of cellular aging [10,11]. It appears that there is a dose–risk relationship between AGEs and adverse health outcomes. In both human and animal models, high levels of accumulated AGEs contribute to type-2 diabetes mellitus (T2DM), obesity, metabolic syndrome, retinopathy, diabetic neuropathy, cardiomyopathy, and kidney disease, as well as a reduced estimated glomerular filtration rate [12,13,14]. A meta-analysis of prospective studies also demonstrated increased risk of mortality from all causes and cardiovascular disease with higher circulating AGEs [15]. AGEs are most commonly implicated in T2DM [16,17,18], due to excess free sugars accelerating the formation of AGEs, in combination with AGEs inducing insulin resistance. Chronic dietary AGE consumption also contributes to arterial stiffness, which can lead to hypertension and systemic inflammation, and may contribute to increased risk of cardiovascular disease in people with T2DM [19,20]. Additionally, while AGEs promote oxidative stress and chronic inflammation and impair insulin sensitivity, hyperglycaemia itself is also a key contributor to AGE synthesis [21].

Due to emerging evidence implicating AGEs in the development and progression of various aging-related diseases associated with brain health (obesity, T2DM), there has been a focus on the neurological sequelae attributed to AGEs, namely neurological and mental disorders. A number of these studies have been conducted recently, adding to the body of knowledge relative to the impact of AGEs in neurological and mental disorders. Therefore, the current review aimed to provide an updated summary of the literature examining the relationship between AGEs and neurocognitive and mental disorders. This information may inform the development of preventative strategies and interventions targeting AGE accumulation.

## 2. Advanced Glycation End-Products (AGEs): Endogenous and Dietary Sources

AGEs are proteins or lipids that are glycated exogenously and endogenously in the presence of reducing sugars, both within the food matrix and in the body, respectively [22]. Specifically, non-enzymatic glycation and oxidation of proteins and lipids occur when they are exposed to aldose sugars [23]. The first reaction in the formation of AGEs leads to a *Schiff base*, which occurs as a consequence of the condensation between a reducing sugar and the free amino group of proteins, lipids, and nucleic acids [24]. This is followed by rearrangement of Schiff bases, leading to the formation of more stable Amadori products. The irreversible formation of AGEs occurs when reducing free sugars attach to the free amino groups of proteins, or through the oxidation of proteins such as elastin, laminin, collagen, lipids, and nucleic acids. Some glycation is an inevitable biochemical process; however, when high methylglyoxal levels are maintained, the development of AGEs can increase [22,25]. The most studied AGE includes N^ε^-carboxymethyl-lysine and the highly reactive derivatives of methyl-glyoxal, argpyrimidine, pentosidine, and methylglyoxal-lysine dimer and glyoxal-lysine-dimer [24]. 

AGEs are ligands for one main receptor, known as the receptor for advanced glycation end products (RAGE) [26]. Upon binding to RAGE, AGEs activate different inflammatory pathways, promoting reactive oxidative species (ROS). This inflammatory response upregulates RAGE, establishing a positive feedback loop [27]. Soluble RAGE (sRAGE) and endogenous secretory RAGE (esRAGE) are found in the circulation, and may function as a decoy receptor by binding pro-inflammatory ligands, such as high mobility group box 1 protein [28] and S100 proteins/calgranulins [29], ultimately preventing them from accessing cell surface RAGE [30,31]. AGEs can also bind to other cell surface receptors such as the AGEs receptors (AGER1, AGER2, and AGER3) [32], which increase the AGE uptake and removal, as well as counteracting AGE-induced oxidative stress [33,34]. The key difference between AGEs receptors and RAGE receptors is that AGEs receptors (AGER1, AGER2) are involved in blocking AGEs–RAGE-mediated intracellular pathologies and degrading AGEs [35]. Conversely, RAGE receptors initiate an inflammatory response and have additional molecules, such as amyloid beta (Aβ) and high mobility group box 1 protein, as their cognate ligans [30]. The body aims to remove the toxicity of the AGEs dicarbonyl precursors via the glyoxalase detoxifying system that prevents the formation of AGEs, and via proteasomal degradation of formed AGEs [26].

The amount of circulating and accumulated AGEs in vivo is influenced by dietary factors [36]. Dietary AGEs are readily absorbed by the gastrointestinal tract. AGEs commonly found in food include N^ε^-carboxymethyl-lysine, N^ε^-1-carboxyethyl-lysine, pyrroline, pentosidine, glyoxal, and methylglyoxal [37,38]. Studies in healthy humans show that dietary AGEs directly correlate with circulating AGEs, such as N^ε^-carboxymethyl-lysine and methylglyoxal [39]. The diet influences endogenous AGE (such as N^ε^-carboxymethyl-lysine, pyrraline, pentosidine) concentrations independently of their consumption. The majority of uncooked food contains low levels of dietary AGEs, particularly fruits and vegetables [40]. However, prolonged high-temperature cooking, particularly with dry heat cooking methods (grilling, roasting), can markedly increase the concentration of AGEs within food, with approximately 10–30% of exogenous AGEs absorbed from the intestine into the blood [37]. Foods commonly consumed with high AGEs include processed foods and cooked foods, particularly those that are fried, roasted, or grilled. The highest AGE containing foods have been reported to be foods processed under high dry-heat conditions including seeds, nuts, and grain products (roast walnuts and sunflowers, crackers, granola), and meats (fried chicken, bacon, beef) [40]. This is largely due to the Maillard reaction, which commonly leads to browning as a consequence of the interaction between the proteins and sugars [41]. Dry heat has the potential to increase the rate of dietary AGE formation by 10 to 100 fold, with higher contributions coming from meats and poultry, due to their highly reactive amino acid and lipids components, which rapidly accelerate AGE production when in the presence of their reducing sugars [8].

Some foods generally recommended as healthy can have high AGE levels, depending on how they are prepared. For example, broiled and fried salmon contain relatively high amounts of dietary AGEs, even though salmon consumption is associated with better overall health and cardiovascular disease outcomes [8,42]. A study by Uribarri et al. tested several cooking methods, finding that higher temperature and lower moisture levels result in higher AGEs (based on N^ε^-carboxymethyl-lysine content) with salmon that is broiled and fried compared to poached. Plant foods that are cooked or charred typically have fewer dietary AGEs compared to animal products, regardless of the cooking method, possibly due to their antioxidant and vitamin content, differences in amino acids, and lower fat content, which can all contribute to less AGE formation [8]. Exceptions include raw chestnuts and sunflower seeds, for example, that contain AGE levels comparable to some animal foods and far greater levels when roasted [8]. Notably, some dietary components, such as plant foods including fruits and vegetables high in natural antioxidants, can reduce the formation of AGEs endogenously [43]. However, vegetarians may have more AGEs than meat-eaters, possibly due to more frequent bouts of elevated blood glucose levels and/or higher consumption of foods and beverages with high fructose-to-glucose ratios [44,45]. 

Based on a review of various studies, a daily average AGE intake between 9000–24,000 kU/day was reported amongst healthy people [36]. However, it has been proposed that these levels are high and pro-inflammatory [24]. At present, there is no clearly established AGE reference range that is associated with chronic illness or health effects. One possible explanation for the high discrepancy between the reported intakes is the use of different diet record methods across studies, as summarized by Nowotny et al. [36]. In addition, many food questionnaires do not include food preparation techniques, with measurement error of the daily average AGE intake remaining possible [36]. Large differences in the precision estimates of AGE concentrations from food sources have also been reported and may depend on the detection method used; i.e., liquid chromatography-mass spectrometry versus enzyme-linked immunosorbent assay. 

A systematic review and meta-analysis of randomized controlled trials showed a reduction in insulin resistance, fasting insulin, and total and low-density lipoprotein cholesterol (LDL-C) in low dietary AGEs groups compared with high dietary AGEs groups [46]. A 24-week randomized controlled trial (n = 62) reported improved lipids (total cholesterol, LDL-C, apolipoprotein B), and a reduction in C-reactive protein and intima-media thickness compared to controls, in a group of people with prediabetes [47]. A smaller eight-week trial (n = 40) in people with metabolic syndrome (MetS) demonstrated a low-energy, low-AGE diet improved central adiposity, and markers of insulin resistance and inflammation [48], while a two week intervention in individuals with body mass index ≥25 showed no effect [49]. Despite promising results overall, more research is needed to establish a cause and effect relationship between exogenously consumed AGEs and their health effects.

## 3. Dietary AGE Pharmacokinetics

Studies reporting urinary excretion, plasma, or tissue accumulation following AGE intake provide insights into the absorption and systemic bioavailability of exogenous AGEs [50]. A number of key factors affect the pharmacokinetics of dietary AGES, including their molecular size, complexity, and interactions between unabsorbed dietary AGEs and gut microbiota [51]. As mentioned, dietary AGEs are readily absorbed by the gastrointestinal tract [50,52]. However, AGE absorption via the gastrointestinal tract is influenced by their chemical characteristics. Dietary AGEs are absorbed in the form of either free-AGEs, low molecular weight peptides (<5 kDa), or protein-bound AGEs [51]. In comparison to low molecular weight peptide bound AGEs (as single amino acids or dipeptides), uptake of free AGEs via the enterocytes apical membrane is relatively low, possibly due to being absorbed via simple diffusion [51]. Uptake and systemic bioavailability of low molecular weight AGEs has been shown to be relatively high, while high molecular mass protein-bound AGEs may be absorbed less efficiently [26,50]. 

High molecular mass protein-bound AGEs require enzymatic digestion before being absorbed and vehiculated into the systemic circulation [50]. Given that many of the high molecular weight AGEs cannot be absorbed in the intestine, they may pass through to the colon and be metabolised by colonic bacteria [52]. Once reaching the colon, AGEs may be processed by the gut microbiota, with some bacteria expressing deglycation enzymes capable of metabolising glycated proteins [53]. Specifically, it has been identified that a number of bacterial taxa (i.e., *Bacillus subtilis*, *Escherichia coli* and *Intestinimonas butyriciproducens AF211*) can partially degrade an AGE precursor (fructoselysine) with a key kinase enzyme [54,55]. For instance, fructoselysine can be converted into fructoselysine 6-phosphate, followed by conversion to lysine and glucose-6-phosphate by *E. coli* [56]. It has also been revealed that intestinal bacteria from healthy adults are able to degrade N^ε^-carboxymethyl-lysine, either partially or entirely, in anaerobic conditions [57]. Some high molecular mass AGEs that are not fully hydrolysed by microbiota-derived enzymes may result in their faecal excretion [50]. As much as 20–50% of ingested N^ε^-carboxymethyl-lysine has been found in faeces [58].

Once absorbed, AGEs are distributed to various tissues, including the liver and kidneys, where they accumulate once they overwhelm the AGE detoxification systems [50]. In addition to renal tissue, the highest AGE levels have been found in the lungs, ileum, and colon of the digestive tract [59]. It is suggested that low molecular weight AGEs can be readily absorbed and cleared by the kidneys and have less opportunity to interact with functional proteins, whereas AGEs derived from digested high molecular weight AGEs can easily accumulate within organs and tissues [51]. Once in the systemic circulation, low molecular mass AGEs are predominantly excreted by the kidneys via glomerular filtration into urine [51]. It has further been shown that free-AGEs and peptide-AGEs can be reabsorbed in the proximal tubule [60]. 

As mentioned, various enzymes and detoxifying systems are capable of degrading intracellular AGEs [26]. Further detoxification is observed via the advanced glycation end product receptor 1 (AGE-R1), which is a macrophage receptor involved in inducing AGE endocytosis and inhibiting the activation of RAGE associated NF-κB [61]. Notably, the elimination and clearance of endogenous AGEs depends on receptor mediated transport into cells, where they can be degraded via lysosomes [50]. 

## 4. AGEs in Neurocognitive and Mental Disorders: Mechanisms of Action

A growing body of evidence implicates AGEs in neurodegenerative diseases such as Alzheimer’s disease (AD) and Parkinson’s disease (PD) [62,63,64], and in mental disorders such as depression and schizophrenia. The results of animal models demonstrate that AGEs can accumulate in the brain alongside tau protein and Aβ [65]. Glycation of these proteins can lead to hyperphosphorylation of tau, and contribute to the stickiness and aggregation of Aβ, both of which are key processes in the pathogenesis of AD [66]. RAGE activation regulates β- and γ-secretase cleavage of amyloid precursor protein, to generate Aβ [67]. Membrane-bound RAGE has also been shown to transport Aβ into the brain [68] by mediating amyloid transport across the blood–brain barrier [69,70]. A mouse transgenic model of AD showed that Aβ binds to RAGE, resulting in transport of Aβ from the bloodstream across the blood–brain barrier [70]. Overexpression of RAGE in neurons or microglia in mice displayed accelerated accumulation of Aβ and exacerbated spatial learning/memory impairment, and neuropathological and biochemical changes [71]. Previous studies found upregulation of RAGE expression in the brains of patients with AD. Receptor-independent AGE peptide cross-linking has also been shown to significantly accelerate the polymerization of Aβ peptides [72]. Moreover, recent in vitro investigations have shown that AGEs induce the aggregation and cross-linking of α-synuclein, a neurofilament protein found in PD [73,74]. 

The presence and accumulation of AGEs may contribute to neuronal cell death and dysfunction, due to their role in the generation of pro-inflammatory cytokines [65]. Recently, dietary AGEs have been shown to directly stimulate the inflammatory response of human innate immune cells [75]. Given that AGEs interact with pro-oxidant RAGE, this results in the generation of free radicals, activation of nuclear factor kappa B (NF-κB), and production of pro-inflammatory cytokines, which lead to cellular dysfunction, such as mitochondrial dysfunction, intracellular ROS, and apoptosis [35,76]. Activation of RAGE results in glial activation, as well as cytokine and ROS production within the brain [77]. AGE production is also intensified in the presence of increased oxidative stress, with excess ROS generating dicarbonyl compounds, by interrupting cellular glycolysis [78]. Mitochondria-induced oxidative stress and inflammation have been observed in AD [79], PD [80], and mental disorders such as depression [81] and schizophrenia [82].

When AGEs accumulate, activated RAGE signalling and production downregulates specific AGE detoxification pathways involving the ubiquitin–proteasome system and autophagy [26,83]. In contrast, when RAGE production is low, the brain can detoxify AGEs such as methylglyoxal, which has strong glycating properties. However, in AD, especially in its later stages and in late-onset AD, the detoxification of AGEs is impaired, due to reduced brain glutathione concentrations [84]. Due to impaired detoxification, AGEs increase ROS production and subsequently upregulate amyloid precursor proteins, Aβ production, and neuronal cell death [85]. One study demonstrated poorer cognitive performance and increased phosphorylated tau and amyloid precursor proteins in mice injected with AGE [79]. The effect of AGEs in the pathogenesis of AD was further confirmed in animal feeding trials, where mice fed a high-AGE diet had poorer spatial learning, higher circulating AGEs and insoluble Aβ_42_, and oxidative stress compared to mice fed a diet low in AGEs [86]. 

Additionally, AGEs inhibit the brain-derived neurotrophic factor (BDNF)-tropomyosin receptor kinase B (TrkB) signalling pathway, thereby impairing neuroplasticity in the rat brain [87]. Wu et al. reported that the intravenous administration of AGEs in rats induced the suppression of BDNF, which in turn mediated tau-hyperphosphorylation in the brain [88]. Impairments in BDNF signalling have been observed in depression and neurodegenerative diseases [89,90]. 

**Figure 1:**
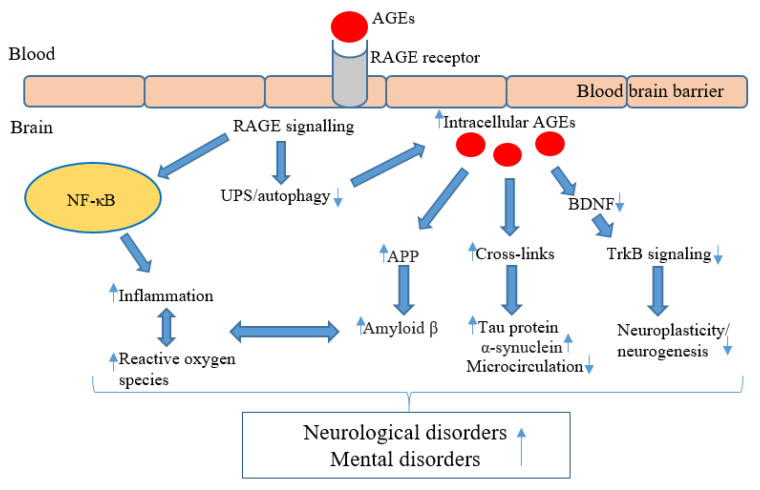
Molecular pathways underpinning the effects of AGEs in the pathogenesis of neurologicognitive and mental disorders. AGEs bind to the RAGE receptor located on the blood–brain barrier, which allows AGEs to enter the brain and to also activate RAGE signalling [98]. RAGE signalling activates nuclear factor kappa B (NF-κB) within glial cells, resulting in the activation of pro-inflammatory cytokines and reactive oxygen species (ROS) production within the brain [77]. The neuroinflammation and ROS support amyloid beta (β) production and promote neuronal cell death [85]. RAGE signalling and production downregulates specific AGE detoxification pathways, involving the ubiquitin–proteasome system (UPS) and autophagy [26,83]. This impaired detoxification further contributes to intracellular AGEs accumulation and upregulating amyloid precursor proteins (APP), leading to amyloid β production [85]. AGEs form cross-links with aggregate tau protein and alpha-synuclein [63,99], in addition to possibly impairing the brain’s microstructure through protein cross-links [91]. AGEs may reduce the brain-derived neurotrophic factor (BDNF) and tropomyosin receptor kinase B (TrkB) signalling pathway, thereby impairing neuroplasticity [87]. ↑ = increase/upregulate, ↓ = decrease/downregulate.

Intracellular accumulation of AGEs results in endothelial dysfunction [91], with the formation of cross-links in the arterial wall and collagen, leading to increased arterial stiffness [92,93]. Endothelial dysfunction and arterial stiffening are also implicated in the development of depressive symptoms and vascular dementia, by impairing microcirculation in the brain and leading to white matter hyperintensities [94,95]. A systematic review investigating the pathological role of AGEs in schizophrenia revealed that alterations in the AGE–RAGE axis may contribute to the vascular diseases commonly comorbid with schizophrenia [96]. Moreover, a Western diet was associated with cardiac hypertrophy, inflammation, mitochondrial-dependent superoxide production, and cardiac AGE accumulation in wild-type mice following a 16-week intervention [97]. Figure 1 summarizes the potential molecular pathways underpinning the ability of AGEs to promote neurological and mental disorders. 

Alternatively, AGEs may play a role in mental disorders indirectly, by affecting a number of conditions that are often comorbid with mental disorders and cognitive impairment, such as obesity, T2DM, and cardiovascular disease [100,101]. AGEs are also related to disease incidence of several organs, such as the liver, kidneys, and heart, through a range of mechanisms [102]. T2DM is associated with greater age-associated cognitive decline, and an increased risk of AD, vascular dementia, and depression [103,104]. It has been postulated that AGEs may be a common pathway contributing to the pathology of both T2DM and dementia [17]. People with T2DM have greater AGE accumulation in the brain [105]. Additionally, people with comorbid T2DM and AD have higher AGE concentrations, greater Aβ, RAGE, tau, and microglial activation than those with AD alone [106]. Systemic inflammation and acute infections can also lead to the production of AGEs in the brain via peripheral AGE-RAGE binding [107]. RAGE is expressed in a wide range of cells, including monocytes, adipocytes, and macrophages, and can bind with other compounds that are implicated in neurological disorders, such as S100β, calgranulin, Aβ, and other fibrillar proteins [3,108]. 

Moreover, preliminary research suggests that dietary AGEs may alter the gut microbiome composition and increase colon membrane permeability [109,110,111]. Specifically, a two-week dietary intervention of Amadori products (AGE precursors) was negatively correlated with bifidobacterial growth in healthy male adolescents [110]. In circumstances of increased intestinal epithelial cell permeability, greater quantities of dietary AGEs may be able to translocate into the systemic circulation, activating host immune responses [78]. These results suggest that AGEs may play a role in neurological function through the gut–brain axis [112,113]. 

## 5. AGEs and Neurocognitive Disorders

Several studies have investigated the association of circulating and tissue AGE levels with cognition [18,114,115,116,117,118,119,120]. Most studies focused on blood AGEs, rather than dietary AGEs or interventions that modify dietary AGEs. 

In a prospective study involving two groups of older people (mean age of 66.0 years, SD = 9.9) with either normal blood glucose levels (n = 425) or T2DM (n = 495), mid and high urine pentosidine levels (a biomarker of AGEs) were associated with lower scores on the digit symbol substitution test at baseline and longitudinally at nine years follow-up, in both groups [105]. The incidence of cognitive impairment was higher in those with high or mid pentosidine levels than those in the lowest tercile (odds ratio = 1.55; 95% CI: 1.07–2.26). Similarly, circulating AGEs have been associated with poorer cognitive performance on the digit substitution test in a cohort of people predominantly diagnosed with T2DM [114]. In the same study, decreased grey matter volume was also observed in people without T2DM with higher AGE consumption. In a cross-sectional study of healthy people (n = 781), higher plasma AGE (pentosidine) concentration was associated with poorer global cognition, with no differences observed between people with T2DM (n = 215) and those without (n = 549) [116]. Furthermore, higher HbA1c levels were associated with both diabetes and long-term cognitive decline in the English Longitudinal Study of Ageing (n = 5189) [121]. Similarly, lower HbA1C and blood glucose levels have been associated with better cognitive performance, in part due to hippocampal volume and microstructure [122]. Moreover, improved glycaemic control and reduced insulin resistance has been associated with reduced endogenous AGE production.

In a sample (n = 3889) of older people (mean age = 72.5 years, SD = 8.9), higher extracellular newly identified RAGE (EN-RAGE) was associated with higher prevalence of dementia [63]. EN-RAGE is an endogenous ligand of RAGE increased in various inflammatory diseases, such as cardiovascular disease and T2DM [123]. Longitudinally (median = 12.4 years), higher levels of EN-RAGE have been associated with dementia prevalence [119]. Higher plasma AGE levels were associated with a poorer Clinical Dementia Scale rating after a 48.6 month follow-up in 25 people with probable AD and T2DM [119]. However, a limitation is that AGEs were only measured at baseline. 

In 144 people with dementia, higher AGE levels were associated with functional mobility and progression to dementia over one year [115]. AGE levels have been investigated in people with mild cognitive impairment and mild dementia in a placebo-controlled trial of benfotiamine, a synthetic version of thiamine that can reduce AGE production [117]. After 12 months, benfotiamine reduced the rate of cognitive decline and AGEs. In older individuals (mean age = 71.0 years, SD = 8.1) with normal cognitive function (n = 49), higher dietary AGEs and serum methylglyoxal were associated with a faster decline in cognitive performance at a 35.9 month (SD = 13.5) follow-up [118]. Table 1 summarises the studies examining AGEs and dementia. These studies, taken together, suggest there is a noteworthy association between AGE levels and cognitive function.

Preliminary research has indicated that there may be a link between AGE accumulation and a number of other neurological disorders, such as multiple sclerosis (MS) and Parkinson’s disease (PD) [124,125]. One study revealed a significant reduction in the soluble receptor for AGEs (sRAGE) amongst people with MS relative to healthy controls [126]. Post-mortem hippocampal slices of patients with MS, AD, and healthy controls demonstrated increased AGE and RAGE in MS patients, resembling those of AD patients [125]. Accumulation of AGEs in PD has been described [127]. A small-scale autopsy study (n = 4) revealed that brain AGE accumulation might, not only be associated with Lewy body cross-linking in the early stages of PD, but also the intracellular oxidative stress that leads to disease progression [74]. One study measured plasma AGEs (N^ε^-carboxymethyl-lysine and N^ε^-1-carboxyethyl-lysine) in patients with PD and AD and healthy controls. In comparison to the controls, higher levels of N^ε^-carboxymethyl-lysine were found in AD and PD patients [124]. 

A six-month randomized pilot trial was conducted to examine the effectiveness of reducing dietary AGE intake in older people with T2DM (n = 75) [18]. Fifty-three of the participants had mild cognitive impairment (MCI) and were randomized between groups. The control arm received standard dietary advice, while the intervention arm received standard dietary advice and advice aimed to reduce dietary AGE intake. The intervention arm was instructed to modify their cooking methods to reduce the temperature and duration of cooking for their food, and when cooking animal products, to avoid dry heat cooking such as frying, baking, or grilling. Instead, they were advised to cook animal products via lower heat cooking methods such as boiling, poaching, stewing, and steaming, all culinary techniques associated with lower production of dietary AGEs. While, no between-group differences in cognition were observed, a reduction in circulating AGE concentrations in the intervention group was reported. Notably, improvements in global cognition were observed in both groups, suggesting that nutritional advice alone benefitted participants independently of AGE intake. 

## 6. AGEs and Mental Health Disorders

### 6.1. Schizophrenia 

A number of studies have indicated that AGEs may be associated with schizophrenia [96,128]. One systematic review revealed that AGE accumulation, in either skin or blood, was elevated in patients with schizophrenia compared to healthy controls [96]. The authors suggested that the relationship between AGEs and elevated oxidative stress was bidirectional and may contribute to schizophrenia pathology [96]. More recent studies have provided further evidence of the relationship between AGE accumulation and schizophrenia. In particular, one study investigated whether AGEs affect cognition in patients with schizophrenia [129]. The results revealed that higher plasma AGEs levels were associated with poorer processing speed in schizophrenia [129]. This inverse association remained robust following the adjustment for a number of confounders, including age and antipsychotic medications.

One case-control study investigated whether skin AGEs were associated with recent-onset psychosis in a cohort of 111 patients [130]. Skin AGE concentration was higher by approximately 15% in comparison to healthy controls (n = 135). This elevated AGE accumulation corresponded to the accumulation of AGEs normally observed in healthy cohorts during an approximately 10-year follow-up [130]. Notably, duration of illness, duration of antipsychotic treatment, and cumulative exposure to antipsychotics were correlated with AGEs. The same authors conducted another longitudinal study that aimed to investigate the accumulation rate of AGEs in recent-onset psychosis, as well as assessing which factors may play a role in the accumulation of skin AGE (ethnicity, tobacco use, cannabis use) [131]. AGE levels were assessed 12–24 months following a baseline measurement in 66 patients and 160 healthy controls. The results demonstrated a significantly higher AGE-accumulation rate in patients who had recent-onset psychosis compared to healthy controls, after adjusting for confounding factors such as cannabis use [131]. In healthy controls, a significant association of AGE-accumulation with ethnicity and tobacco exposure was found. Another longitudinal study reported that skin AGE concentrations may predict the risk of persistent psychotic symptoms (odds ratio = 1.68, CI = 1.05–2.69) in drug-naive adolescents over a 12-month follow-up period [128]. These findings were suggestive of the involvement of AGEs in the pathophysiology of early psychosis.

Hammoudeh et al. examined AGE concentrations in participants with chronic mental disorders while taking antipsychotics for at least six months [132]. Results showed there was no difference in levels of skin AGEs among participants taking antipsychotics compared with a control group from the general population. These findings indicated elevated AGE concentrations may relate to disease severity, irrespective of medication intake.

A recent cross-sectional study assessed the effects of AGEs on the volume of various brain regions in patients with recent-onset psychosis (n = 46; mean age = 23.7 yrs) [133]. Magnetic resonance imaging (MRI) showed a significant negative association of skin AGE levels and hippocampal, posterior cingulate gyrus and superior temporal gyrus volumes. These findings point to the potential neuropathological link between oxidative stress and AGEs in schizophrenia. 

The relationship between pre- and post-operative sRAGE and delirium was examined following cardiac surgery [134]. Delirium is accompanied by symptoms such as psychosis, confusion, and depression. Low baseline antioxidant capacity was independently associated with postoperative delirium development. Interestingly, pre- and postoperative antioxidant capacity levels were negatively correlated with postoperative sRAGE concentration. Given that sRAGE plays an important role in suppressing RAGE signals that induce pro-inflammatory gene activation [135], these preliminary results suggest that sRAGE (and therefore AGEs) may be involved in post-operative delirium that is mediated by antioxidant capacity. 

### 6.2. Depression/Affective Disorders

Associations between AGE and other mental disorders such as depression and affective disorders have also been observed. A large scale cross-sectional study (n= 862) investigated the association of skin and plasma AGEs with depressive symptoms and depressive disorder [136]. The authors also examined whether the potential association was present for somatic (sleeping problems, such as insomnia or hypersomnia, and fatigability; and appetite problems, such as suppressed appetite or excessive eating) and/or cognitive symptoms of depression (i.e., lack of interest, depressed mood, concentration problems, psychomotor agitation/retardation). Higher skin AGEs were associated with depressive symptoms and depressive disorder after adjustment for age, sex, T2DM, smoking, body mass index (BMI), and kidney function. In addition, pentosidine (plasma AGE) was associated with somatic symptoms only, while N^ε^-carboxymethyl-lysine and N^ε^-1-carboxyethyl-lysine were not independently associated with depressive outcomes.

A cross-sectional association was examined between skin AGEs and a number of affective disorders, which included major depressive disorder (n = 1702), dysthymia (n = 828), generalized anxiety disorder (n = 3313), panic disorder (n= 2345), and social phobia (n = 691) [137]. Total elevated AGEs were associated with the presence of all affective disorders, after adjusting for socioeconomic status, but not cardiometabolic factors (metabolic syndrome) or somatic morbidities (i.e., malignancy, kidney disease, etc.) (OR = 1.09, CI: 1.07–1.12). Notably, the strongest association was observed for major depressive disorder and was independent of sociodemographic status, cardio metabolic factors, and somatic morbidities (OR = 1.31, CI: 1.25–1.36). Using this data, the same authors conducted a five-year prospective study to examine the association between skin AGEs at baseline and incidence and persistence/recurrence of affective disorders at follow-up [138]. Elevated AGEs significantly raised the odds of incident affective disorders, most prominently for major depressive disorder (OR = 1.11, CI: 1.04–1.19). However, there was little evidence of an association after adjustment for socioeconomic status, and there was no evidence of an association between AGEs and persistent/recurrent depression, regardless of adjustment. These results suggest that the prospective association of AGE-accumulation with common mental disorders may not be independent of sociodemographic factors. 

A large cross-sectional investigation involving a cohort derived from the Helsinki Birth Cohort Study [139] measured skin AGE concentrations in conjunction with specific depressive symptoms. The highest crude AGE levels were found in those with melancholic depressive symptoms, followed by those with non-melancholic depressive symptoms and those with no depressive symptoms. Interestingly, men had both higher AGE levels and comorbidity indexes (based on the Charlson Comorbidity Index, which assesses 19 conditions) than women, but lower rates of depressive symptoms. These results point to the importance of considering depression type (melancholic vs. non-melancholic) as well as gender when examining the association between AGEs and depression. 

Another study investigated whether skin AGE concentrations mediated the association between affective disorders and excess mortality (n = 81,041) [140]. Mortality was increased in cases with major depression compared to healthy controls. For major depression, mediation by AGEs (5.5–10.3%) was largest, and remained significant after adjustment for sociodemographic and health-related factors. 

Several studies examined levels of soluble RAGE and depression [141,142]. One exploratory pilot study established significantly lower serum sRAGE levels amongst patients with major depression in comparison to a healthy control group [141]. A study focusing on hospitalized patients revealed a negative correlation between serum endogenous secretory RAGE (esRAGE) levels and depression in those diagnosed with T2DM [142]. This suggested lower concentrations of anti-inflammatory esRAGEs in T2DM and, therefore, an increased AGE–RAGE interaction that may be associated with depression. 

There are also a number of studies that failed to demonstrate a link between AGEs and mental disorders. In one small-scale cross-sectional study that involved a cohort of individuals with clinically diagnosed bipolar disorder (10 euthymic, 12 depressed, 13 manic) and 10 healthy control subjects [143], lower lymphocyte AGE concentrations were evident in bipolar patients compared to healthy subjects, while depressed patients displayed higher concentrations of markers indicative of neuronal injury (two-fold higher S100B levels) but not AGEs compared to controls. Notably, AGEs were measured in leukocytes, with the authors attributing the findings to an oxidative stress-induced AGE degradation or low intracellular sugar levels, which are common in bipolar disorder. Another study measured skin AGE concentrations in groups of patients with schizophrenia (n = 27), depression (n = 26), dementia (n = 10), and healthy controls (n = 26) [144]. Although the group with neurocognitive disorders presented with the highest AGEs levels, concentrations were not different between groups, or when compared to a healthy control group. However, the number of participants was relatively small, and the severity of clinical symptoms were not assessed, due to all participants presenting with mild symptoms. Table 2 summarises the studies examining AGEs and mental disorders.

## 7. Limitations and Future Directions

The current evidence provides preliminary support for the association between elevated AGEs levels in the pathology of cognitive and mental disorders. However, several limitations are present that require further investigation. Studies involving neurological disorders have been predominantly centred around dementia, and those focusing on mental disorders have focused on schizophrenia and depression. Although a number of large scale population-based studies examined associations between AGEs and these conditions, participants of non-Western descent were underrepresented, who may display varying amounts of AGEs as a result of varying race or socioeconomic status [130,145]. Cross-sectional investigations largely explored the associations between AGEs and mental disorders. This association is likely multi-factorial, and directionality and causality cannot be inferred based on these cross-sectional studies. Future prospective studies that examine the long-term accumulation of AGEs, as well as nutritional interventions, are needed to determine the effects of AGEs on the trajectory of cognitive and mental disorders. 

While a number of studies utilized clinically defined populations, others measured cognitive function and depressive symptoms or relied on self-reported measures in non-clinical populations. The severity of depressive symptoms may dictate the strength of associations with AGEs, particularly given that those with clinical depression may be diagnosed with related comorbidities that are linked to AGE accumulation. This association may be influenced by the type of depressive symptom examined, with one study demonstrating the highest AGE levels in those with melancholic depressive symptoms compared to other depressive symptoms [139]. Examining the effects of routine psychiatric medications on AGE concentrations in clinically diagnosed cohorts may provide insights into treatment effectiveness and mechanisms of action. In addition, it remains unclear whether there is a bidirectional relationship between AGE accumulation and the pathways implicated in neurological or mental disorders. For example, bidirectional associations have been demonstrated between AGEs and inflammatory processes, with the latter heightened in neurocognitive and mental disorders [146,147,148]. 

A number of key confounders, including sociodemographic status [138] and gender [139], may impact the relationship between AGEs and cognition or mental disorders and ought to be considered in future investigations. Biological factors that are known to increase AGE accumulation, such as aging [16], and commodities, such as diabetes [16] and osteoarthritis [149], should also be considered when assessing the association with depression and cognition. Environmental factors, such as exposure to cigarette smoke and pollution, may further influence AGE concentration and its link with cognition and mental disorders [96].

Recent studies have suggested that the ratio of AGEs and sRAGE is a more accurate biomarker for age-related diseases than either alone [150], with a higher ratio of AGEs to sRAGE exerting more adverse health outcomes. Future studies measuring neurological and mental disorders are encouraged to consider measuring this ratio. Another factor to consider for future studies is the method used for measuring AGEs. Although AGEs in the skin can be measured reliably and non-invasively [151], it is crucial to establish whether specific circulating AGEs (as a more invasive measure) provide different levels of precision. That is, it remains unknown whether skin versus blood AGEs are context-specific and correlate more strongly with cognitive and mental disorders. Although skin autofluorescence has been shown to correlate strongly with plasma circulating AGEs [152], research suggests that measuring circulating AGEs may be ideal for short interventional studies, and measuring tissue AGEs and skin autofluorescence for screening or long-term interventional studies [93]. 

With regard to studies involving dietary AGEs, no validated questionnaires currently exist that enable the accurate estimation of dietary AGE intake at the population level [78]. Moreover, although the lowest possible intake of dietary AGEs has been recommended for optimal health (<9000 kU/day) [34], it remains unclear exactly how much dietary AGEs can be consumed and for how long, before their health effects are noticed. 

**Figure 2:**
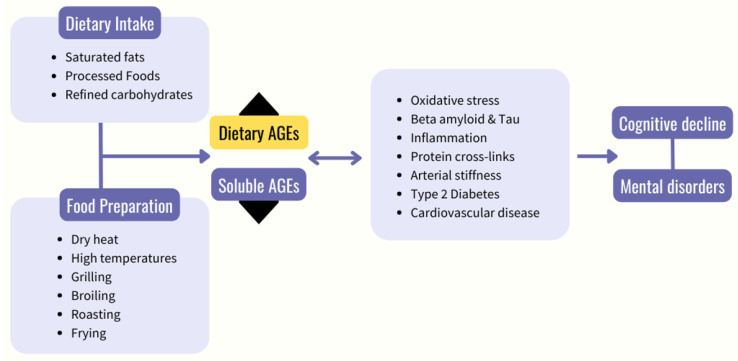
Proposed pathway through which dietary AGEs contribute to cognitive decline and mental disorders. Black arrow pointing up indicates an increase in dietary advanced glycation end products (AGEs), and black arrow pointing down indicates a decrease in soluble AGEs.

Future studies need to examine whether AGE concentration is a worthwhile target for interventions aimed at alleviating neurological or mental disorders. Similarly, this may prompt studies to better establish the risk factors that may be associated with mental and neurological disorders through the accumulation of AGEs, such as diets high in ultra-processed foods [16]. A systematic review, based on twelve dietary AGE interventions with a total of 293 participants, indicated that a high AGE diet compared to a low AGE diet may contribute to risk factors associated with chronic disease, such as inflammation and oxidative stress [153]. However, there were discrepancies in the AGE concentrations that constituted high vs. low between trials. There is currently limited exploration into the effects of a low dietary AGE intervention on cognitive function [18], with no studies exploring the effects of dietary AGEs on mental disorders. Ideally, direct comparisons between low vs. high AGE dietary interventions would provide the most insight. This is supported by recent evidence highlighting a significant reduction in a series of conditions that are often comorbid with mental and neurological disorders (insulin resistance, elevated fasting insulin, and high total and LDL-C) following a low versus high AGE diet [46]. Figure 2 outlines the proposed pathway by which dietary AGEs may impact cognition and mental health.

The link between AGEs and T2DM is well recognized; however, the interplay between the two and risk of neurocognitive disorders and mental disorders requires further investigation. As mentioned previously, AGE accumulation represents a common contributing factor to both T2DM and neurocognitive disorders, such as AD and its associated pathologies [154]. Moreover, diabetic hyperglycaemia also contributes to endogenous AGE formation, which is suggestive of a possible bidirectional link between AGEs and T2DM [155,156]. Inflammatory processes associated with AGE consumption and accumulation that are common in neurocognitive disorders could be targeted through low-AGE diet and low glycaemic index dietary interventions. However, evaluating the effectiveness of dietary interventions in the prevention of neurocognitive disorders is inherently difficult, due to the expense and difficulty in performing long-term controlled trials. Given that a reduction in serum AGE concentrations is accompanied by a simultaneous reduction in markers of inflammation, oxidative stress, and endothelial dysfunction [157], nutrients that target these pathologies may be promising interventions to alleviate the effects of AGEs. Several food bioactive derivatives such as flavonoids [158], dietary antioxidants (alpha-tocopherol) [159], pyridoxamine [160], thiamine [161], fruits and vegetables [43], and molecules with anti-glycation properties [162] may inhibit AGE formation, as well as counteract the pathophysiological mechanisms triggered by AGEs, including inflammation and oxidative stress. A recent review suggested that the Mediterranean diet could provide a model for the reduction of dietary AGEs [163]. Consumption of foods with a low-glycaemic index that are naturally low in AGEs, including those derived from dietary patterns such as the Mediterranean and DASH (dietary approaches to stop hypertension) diet, have demonstrated potential for reducing cognitive decline and the incidence of mental disorders, particularly in those with T2DM [164,165,166]. As a result, healthy dietary interventions may represent affective strategies for avoiding the detrimental effects of AGE accumulation. Further strategies such as incorporating ingredients including citrus and/or vinegar into cooking methods or meals may also inhibit or reduce AGE formation [167]. Although many of these compounds have the potential to inhibit the formation of AGEs, further research focusing specifically on dietary endogenously-produced AGEs in mental and neurological disorders is warranted.

## 8. Conclusions

The reviewed literature indicates that elevated circulatory and skin AGEs are associated with neurological disorders, especially dementia, and mental disorders, such as depression and schizophrenia. Longitudinal investigations have revealed that elevated AGEs may increase the risk of cognitive impairment, which appeared to occur independently of metabolic risk factors. In addition, limited but supporting data show that higher skin AGE accumulation may be associated with depression and schizophrenia. Elevated oxidative stress and neuroinflammation are key pathophysiological mechanisms linking AGEs with impaired brain health. This paradigm is also supported by the close relationship between AGEs, T2DM, and cardiovascular disease, which in turn are strongly implicated in the pathogenesis of neurocognitive diseases and mental disorders. However, given that the majority of studies have largely relied on cross-sectional designs, with a notable lack of experimental research, directionality and causal pathways in the relationship between dietary sources of AGEs and cognitive and mental disorders remain unknown. Future studies are encouraged to address these gaps.

## Figures and Tables

**Table 1:** Studies assessing the association between AGE concentrations and dementia.

Study	Study Design	Sample Size	Age (Mean ± SD) Years	Participant Sex (Male)	AGE Measure	Results
Adams et al., 2017	Cross-sectional	816	66.0 ± 9.9	46.6%	Serum AGE	Higher AGEs were associated with poorer digit symbol substitution test performance and decreased grey matter volume.
Chen et al., 2021	Longitudinal	3889	72.5 ± 8.9	43.8%	Skin AGE, Plasma EN-RAGE & S-RAGE	At baseline, higher EN-RAGE associated with higher prevalence of dementia, whereas higher S-RAGE associated with a lower prevalence. After 12.4 years on average, only EN-RAGE was associated with dementia prevalence.
Chou et al., 2019	Longitudinal	25	79.0 ± 5.8	12%	Plasma AGE	Higher AGEs were associated with a decline in the CDR after a 48.6 ± 2.1 month follow-up in people with AD and T2DM.
Drenth et al., 2017	Longitudinal	144	80.7 ± 7.7	43.7%	Skin AGE	Functional ability was associated with AGE levels and dementia progression over one year.
Lotan et al., 2021	Randomized control trial	75	Intervention: 71.9 ± 4.29 Control: 71.42 ± 3.99	Intervention: 77.1% Control: 72.5%	Serum AGE	Reduced dietary AGE intake and standard dietary advice improved cognitive performance in people with T2DM. More improvement was observed in people with MCI in the intervention group.

Legend: CDR = clinical dementia rating; esRAGE = endogenous secretory RAGE; GAD = general anxiety disorder; NA = not assessed; MCI = mild cognitive impairment; sRAGE = soluble receptor for advanced glycation end product.

**Table 2:** Studies assessing the association between AGE concentrations and mental disorders.

Study	Study Design	Mental Disorder	Sample Size	Age (Mean ± SD)	Participant Sex	AGE Measure	Results
Chen et al., 2012	Cross-sectional	Depression	71	57.39 ± 9.80	37% male	esRAGE	Inverse correlation between esRAGE levels and depression in those diagnosed with T2DM.
Emanuele et al., 2011	Cross-sectional	Schizophrenia, depression,	148	48.4 ± 11.6	40% male	sRAGE	Significantly lower serum sRAGE levels amongst patients with major depression in comparison to a control group.
Errikson et al. 2021	Cross-sectional	Depression	815	76	43.8% male	Skin AGEs	The highest AGEs levels were found in those with melancholic depressive symptoms, followed by non-melancholic symptoms
Hammoudeh et al., 2017	Cross-sectional	Bipolar disorder (41%), schizophrenia, depression	48	35.8 ± 10.1	Na	Skin AGEs	No differences between higher AGEs levels among patients on antipsychotics compared with the controls.
Hagen et al., 2017	Case-control	Recent onset psychosis	532	Na	Na	Skin AGEs	Patients with a recent onset of psychosis had increased AGEs levels compared to healthy controls.
Hagen et al., 2020	Prospective	Recent onset psychosis	238	26.6	78.8% male	Skin AGEs	Increased AGE-accumulation rate was shown in recent onset psychosis compared to healthy controls
Hagen et al., 2020	Cross-sectional	Depression, dysthymia, GAD, panic disorder, social phobia	81,041	44.1 ± 12.3	41.7% male	Skin AGEs	The strongest association between AGEs and affective disorders was observed for major depressive disorder, after controlling for sociodemographic, cardio metabolic factors, and somatic morbidities.
Hagen et al., 2020	Prospective	Depression, dysthymia, GAD, panic disorder, social phobia	43,267	42.2 ± 10.4	41.8%	Skin AGEs	Elevated AGEs significantly raised the odds of incident affective disorders, most prominently for major depressive disorder. Incidence was reduced after adjusting for socioeconomic status.
Hagen et al., 2021	Prospective	Depression, dysthymia, GAD, panic disorder, social phobia	81,041	Na	Na	Skin AGEs	In major depression, mortality was most largely mediated by AGEs.
Kabori et al., 2021	Cross-sectional	Schizophrenia	58	46.8 ± 11.4	71% male	Plasma	Processing speed was associated with AGEs.
Kaźmierski et al., 2021	Prospective	Post-operative delirium	177	67	78% male	sRAGE	Both pre- and post-operative sRAGE levels were increased in patients who developed delirium compared to non-delirium patients
Miyashita et al., 2021	Prospective	Psychosis	282	13.4 ± 0.6	55.3% male	Skin AGEs	Fingertip AGEs potentially predicted the trajectory of psychotic symptoms among drug-naive adolescents over 12 months.
Moutsatsou et al., 2014	Cross-sectional	Bipolar disorder	45	44.6 ± 3.9	40% male	Leukocyte AGEs	Lower lymphocyte AGE concentrations were displayed in bipolar patients compared to healthy controls.
van Dooren et al., 2017	Cross-sectional	Depression	862	59.8 ± 8.5	55% male	Skin and plasma AGEs	Higher skin AGEs were associated with depressive symptoms and depressive disorder. Pentosidine was associated with somatic symptoms only.
Yamashita et al., 2020	Cross-sectional	Depression, schizophrenia	87	55.3 ± 7.8	33% male	Skin AGEs	A mental disorder diagnosis did not significantly affect the skin AGEs in comparison to a healthy control group.

Legend: esRAGE = endogenous secretory RAGE; GAD = general anxiety disorder; NA = not assessed; sRAGE = soluble receptor for advanced glycation end product.
